# Evaluation of probiotic growth stimulation using prebiotic ingredients to optimize compounds for *in ovo* delivery

**DOI:** 10.3389/fmicb.2023.1242027

**Published:** 2023-09-22

**Authors:** Niloofar Akhavan, Katarzyna Hrynkiewicz, Dominika Thiem, Cinzia Randazzo, Ann M. Walsh, Kieran J. Guinan, John T. O’Sullivan, Katarzyna Stadnicka

**Affiliations:** ^1^Department of Microbiology, Faculty of Biological and Veterinary Sciences, Nicolaus Copernicus University, Torun, Poland; ^2^Faculty of Health Sciences, Nicolaus Copernicus University in Toruń, Ludwik Rydygier Collegium Medicum, Bydgoszcz, Poland; ^3^Department of Agriculture, Food and Environment, University of Catania, Via Santa Sofia, Catania, Italy; ^4^BioAtlantis Ltd., Tralee, Ireland

**Keywords:** probiotics, prebiotics, synbiotics, poultry breeding, selection of prebiotics

## Abstract

The use of probiotics, prebiotics and synbiotics in poultry diets beneficially stimulates the gut microbiome thus promoting the health and welfare of the animals. In this study, we analyzed 7 poultry probiotics (*Lactobacillus plantarum* – B1 and B4, *Lactobacillus rhamnosus* – B3, *Bifidobacterium lactis* – B2, *Carnobacterium divergens* – B5, *Propionibacterium thoenii* – B6, *Clostridium butyricum* – B7) and 12 prebiotics, differing in chemical composition and source of origin (fungi, algae, animal, etc.). The main goal of our research was to select the most promising candidates to develop synbiotic combinations. We determined the growth kinetics of all probiotics in the presence of prebiotics in a series of *in vitro* studies to select optimal combinations. Five out of seven investigated probiotics were significantly stimulated by astragalus polysaccharide, and this prebiotic was characterized in our work as the most effective. Moreover, in the case of three probiotics, B2, B3 and B4, significant growth stimulation has been found when beta-glucan, vegetable protein hydrolysate and liquid seaweed extract were supplied. Strain B1 (*L. plantarum*) was stimulated by 6 out of 12 prebiotics. The growth of B4 (*L. plantarum*) and B2 (*B. lactis*) was enhanced by prebiotics after 2 h of incubation. A high growth rate of 3.13% was observed in the case of *L. plantarum* (B4) and a 3.37% higher rate for *B. lactis* (B3), compared to the growth of probiotics in the control medium with glucose but no prebiotics. The best candidates for synbiotic combinations based on this *in vitro* work are the strains belonging to *L. plantarum* (B4), *L. rhamnosus* (B3) and *B. lactis* (B2), consistent with prebiotics such as astragalus polysaccharides and vegetable protein hydrolysate. These combinations will be subject to future *in vivo* poultry trials involving the *in ovo* microbiome modulation.

## Introduction

Research suggests that probiotics have a beneficial role to play in the management of gastrointestinal diseases in animals. Likewise, prebiotics regulate intestinal microbiota and create a shield against damage to the intestinal barrier (Brugman et al., 2018). Prebiotics are increasingly utilized in dietary strategies aimed at enhancing animal and human health, given their potential antibacterial and antioxidant properties, as well as their ability to influence the gut microbiota by promoting the growth of beneficial bacteria (Brugman et al., 2018). *In vitro*, testing of the growth kinetics of probiotics with prebiotics is required to assess the compatibility of the combined formulation to develop synbiotics.

Among probiotics, *Lactobacilli* and *Bifidobacterium* might be used to enhance animal health, growth performance and in consequence, food safety. Strains with unique and specific characteristics that may provide health advantages could potentially be developed from the animal gut, soil environment and dairy. The use of different *Bifidobacterium* strains (such as *B. animalis*, *B. longum* or *B. infantis*) as probiotics for poultry has been shown to increase body weight and reduce total coliform (Abdel-Moneim et al., 2020).

The use of prebiotics in poultry increases the quality of poultry gut microbiome. Prebiotics regulate the development of Bifidobacteria, inhibit the growth of harmful microorganisms and eliminate toxic substances. Prebiotics also affect the synthesis of vitamins such as nicotinic acid, B1, B2, B6, B12, or folic acid (Yaqoob et al., 2021). *Bifidobacterium* has an antibacterial effect because it can inhibit potential pathogens in the intestine of poultry. Fructo-oligosaccharides (FOS) are well-known, commercial prebiotics that stimulate the growth of *Bifidobacterium* in the intestine of chickens, potentially as it reaches the large intestine (where it can affect *Bifidobacterium*) and are not digested in the short intestine (Hajati and Rezaei, 2010). *Lactobacillus* strains have a long history of use as modulating factors for the poultry gastrointestinal tract (GIT), due to their good survivability and biosafety (de Vries et al., 2006). Feeding chicken with *Carnobacterium divergens* in a mixture with other probiotics led to the minimization of the degree of *Campylobacter* spp. There are few studies on the potential of *Propionibacterium thoenii* as a probiotic. This group of probiotics can attach to epithelial cells, live in acidic conditions and the presence of bile salts, and have modest antibacterial activity against *E. coli* (Campaniello et al., 2015). In the study of Yang et al. (2012) the application of *Clostridium butyricum* led to a similar animal performance as compared to the antibiotic colistin sulfate, which was used as a growth-promoting agent.

Generally acknowledged prebiotics for livestock animals include inulin-derived fructo-oligosaccharide (FOS) and inulin products. Examples of widely used prebiotics in the poultry industry are Mannan-Oligosaccharides (MOS), XOS (Xylo oligosaccharides), malto-oligosaccharides, galacto-oligosaccharides (GOS), glycol-oligosaccharides, pectin, gluco-oligosaccharides, lactose, and its derivatives (lactosucrose and lactulose) according to (Yaqoob et al., 2021). Synbiotics are mixtures of prebiotics and probiotics that synergistically act to enhance host performance by boosting the survivability and productivity of beneficial bacteria in the gastrointestinal tract. However, due to the interdependent manner of action of the synbiotic and microbiome compounds, there are still gaps in knowledge on the detailed mechanisms of activity in hosts. This knowledge is required for “diagnosis” of the host gut status leading to “prescription” of the required bioactive ingredients to improve the status quo (Tletseruk et al., 2022). Finding the optimal combinations of bioactive ingredients for synbiotics is therefore challenging and requires more research. In some areas, especially in the prenatal (*in ovo*) application, the EU regulations to facilitate the registration of novel compounds are in place, but with no marketed products as yet. *In vitro* studies allow for the assessment of probiotic growth in combination with different bioactive components. Therefore, an *in vitro* testing stage is proposed as a practical tool to verify and characterize the efficacy of candidate probiotics and prebiotics before injecting them *in ovo*. An *in vitro* testing stage is also proposed for dosage optimization and for further prenatal trials aimed at influencing gut and immune system development for the long term and trials to assess post-hatch effects. In this study, an *in vitro* synbiotic optimization trial was undertaken to gain a more thorough understanding of the activity of the candidate probiotics from the groups of *Lactobacillus* sp.*, Bifidobacterium* sp.*, Carnobacterium* sp.*, Propionibacterium* sp. and *Clostridium* sp. with the twelve prebiotic candidates. Results may indicate which prebiotic components have the potential to significantly increase the growth of probiotics. The outcome is the generation of a list of the candidates for synbiotic compositions and the provision of a study protocol to develop new formulations of synbiotics. Based on these findings, the optimal synbiotic compositions are proposed for *in ovo* inoculation and *in vivo* tests, in further research.

## Materials and methods

### Selection of probiotic strains and prebiotics for synbiotic formulations

Seven different probiotic strains originally isolated from poultry and humans were chosen based on the review of multiple research papers focused on probiotics beneficial to poultry and humans. The specific criteria employed to select the bioactive compounds were: specificity for the target host species (at least some of the candidate pro/prebiotics should be previously isolated from the chicken or with the prior demonstration of proven beneficial effect in the chicken), specificity for *in ovo* application (e.g., the prebiotic compound must be fully soluble in water and physiological saline), the novelty of the compound for *in ovo* application (examples of compounds that have not been previously investigated for the purpose of *in ovo* delivery). Further technical criteria applied in the selection included the following: (1) whether the pro/prebiotic compound was previously used as a synbiotic compound; (2) whether the compounds effects have been reported in previous poultry research (data on body weight, feed conversion ratio, gastrointestinal health, immunological parameters or changes associated with the immune system or gut integrity); (3) the form of a product: lyophilized compound, or liquid; (4) earlier reported effects on microbiota: whether the growth of beneficial populations have been stimulated, i.e., at least Lactobacillaceae and Bifidobacteriaceae; (5) earlier reported effects on reducing pathogenic species; (6) source of purchase or delivery to ensure the stable deliveries during pandemics and continued access for potential further *in vivo* trials; (7) for the prebiotic we also applied this criteria to ensure the diversity of candidates to develop the repeatable *in vitro* testing guide. The introduction to this concept is published by (Siwek et al., 2018; Tavaniello et al., 2019).

Culturing of each probiotic was performed for 24 h. Five bacterial strains B1 (*Lactobacillus plantarum* ATCC 11974), B2 (*Bifidobacterium lactis* NCC2818), B3 (*Lactobacillus rhamnosus* H25), B4 (*Lactobacillus plantarum*), and B6 (*Propionibacterium thoenii*) were cultured anaerobically in MRS broth (De Man, Rogosa, Sharpe; Millipore) at 37°C in carbon dioxide incubator (5% CO_2_). Probiotic B5 (*Carnobacterium divergens*) was cultured in Tryptic Soy Broth (TSB) and B7 (*Clostridium butyricum* ATCC 19398) on MRCM (Modified Reinforced Clostridial Broth; https://www.atcc.org/products/860) under the same conditions. B1, B2 and B7 strains were obtained from ATCC. Strains B3 and B4 were received under the cooperation of the research institutions which the co-authors of this study are affiliated with. Strains B5 and B6 had been isolated and described by the JHJ company in Poland. Strains *L. plantarum, C. divergens*, and *P. thoenii* were characterized in this work (description below). All of these strains are stocked in the Microbiology Department of Nicolaus Copernicus University at −80°C.

The prebiotics and probiotics used in our study were selected based on a follow-up review and conclusions from the previous in-house research of the co-investigators of this study and based on the available scientific literature. Among all these prebiotics, only 12 prebiotics (ab. 50%) passed the solubility and stability test, which is described further in this section. Twelve prebiotics were used in the experiment for synbiotic design: P1 – Beta-glucan (BioAtlantis, Ltd., Tralee, Ireland), P2 – Vegetable protein hydrolysate (BioAtlantis, Ltd., Tralee, Ireland), P3 – Liquid seaweed extract (BioAtlantis, Ltd., Tralee, Ireland), P4 – Standard inulin, P5 – Long-chain inulin (BENEO GmbH; Polish distributor: Hortimex), P6 – Raffinose (Sigma Aldrich/Merck Group), P7 – Galactooligosaccharides (GOS) (Clasado Biosciences, Ltd. United Kingdom), P8 – Snow crab-derived chitooligosaccharides (Guangzhou Youlan Marine Biotechnology Co., Ltd.), P9 – Saccharicter-penin (Hubei Widely Chemical Technology Co., Ltd.), P10 – Lentinus, P11 – Mannan oligosaccharides, P12 – Astragalus polysaccharide (Xi’an Weizhen Biotechnology Co., Ltd.) (Table 1).

**Table 1:** Prebiotics and probiotics chart names and symbols.

Symbol	Prebiotics name	#	Probiotics name	#	#
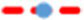	Ctrl		*Lactobacillus plantarum* ATCC 11974	B1	a
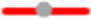	Ctrl + Glu				
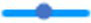	Beta-glucan	P1	*Bifidobacterium lactis* NCC2818	B2	b
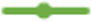	Vegetable protein hydrolysate	P2			
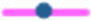	Liquid seaweed extract	P3	*Lactobacillus rhamnosus* H25	B3	c
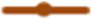	Standard inulin	P4	*Lactobacillus plantarum*	B4	d
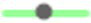	Long chain inulin	P5	*Carnobacterium divergens*	B5	e
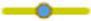	Raffinose	P6	*Propionibacterium thoenii*	B6	f
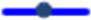	Galactooligosaccharides (GOS)	P7	*Clostridium butyricum* ATCC 19398	B7	g
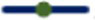	Snow crab-derived Chitooligosaccharides	P8			
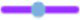	Saccharicter-penin	P9			
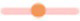	Lentinus	P10			
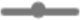	Mannan oligosaccharides	P11			
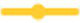	Astragalus polysaccharides	P12			

### Molecular identification of three probiotic bacterial isolates

Three probiotics (B5, B6, B7) isolated and provided by JHJ company were molecularly characterized in this work. DNA isolation was performed by GeneMATRIX environmental DNA extraction kit (EurX, Poland) following the manufacturer’s protocol. Universal primers for 16S rRNA: 27f (5′-AGAGTTTGATCMTGGCTCAG-3′) and 1492r (5′-TACGGYTACCTTGTTACGACT-3′) (Szymańska et al., 2021). Amplification products were resolved by agarose gel electrophoresis (1%) and visualized using a gel documentation system (UVP, MultiDoc-It™ System). The PCR products were purified with a PCR/DNA Clean-up purification kit (GenoPlast Biochemicals, Poland). The concentration and quality of amplicons were measured spectrophotometrically using Nanodrop (Thermo Scientific™NanoDrop 2000). The samples were confirmed on 1% agarose gel electrophoresis. The final DNA products were sequenced at the Institute of Biochemistry and Biophysics Polish Academy of Sciences (IBB, Warsaw, Poland). The forward and reverse sequences were merged and contigs were analyzed by Sequencher 5.4.6. Analyzed strains were identified as B5 – *C. divergens* (Acc No: OP804339), B6 – *P. thoenii* (Acc No: OP804341), B7 – *L. plantarum* (Acc No: OP804340) (Table 2).

**Table 2:** Prebiotics chart name and their sources.

Code	Prebiotic name	Origins
Ctrl	Control	–
P1	Beta-glucan (F981M156A)	BioAtlantis, Ltd., Ireland
P2	Vegetable protein hydrolysate (B001P335)	BioAtlantis, Ltd., Ireland
P3	Liquid seaweed extract (A114P252)	BioAtlantis, Ltd., Ireland
P4	Standard inulin	BENEO GmbH; Polish distributor: Hortimex
P5	Long chain inulin	BENEO GmbH; Polish distributor: Hortimex
P6	Raffinose	Sigma Aldrich/Merck Group
P7	Galactooligosaccharides (GOS)	Clasado Biosciences, Ltd. (UK)
P8	Snow crab-derived chitooligosaccharides	Guangzhou Youlan Marine Biotechnology Co., Ltd
P9	Saccharicter-penin	Hubei Widely Chemical Technology Co., Ltd
P10	Lentinus	Xi’an Weizhen Biotechnology Co., Ltd
P11	Mannan oligosaccharides	Xi’an Weizhen Biotechnology Co., Ltd
P12	Astragalus polysaccharides	Xi’an Weizhen Biotechnology Co., Ltd

### Prebiotic preparation

To ensure the stability of inoculation environments for probiotics, only those prebiotics that were demonstrated to be soluble in water and in the growth media were selected to proceed to the main experiment. The solubility test was performed at four temperatures: 4°C, 30°C, 40°C, and 85°C, with moderate mixing at 400 rpm. All media preparations containing prebiotics were filtered separately before use in the experiment. Liquid or powder prebiotics were added to the respective broth media to the final concentration of 2% (w/v) for powder prebiotics and 2% (v/v) for liquid prebiotics and then filtered using Titan3™ PES (polyethersulfone) Syringe filters (0.22 micrometer; Alchem, Poland) to prevent contamination.

### Cultivation of probiotics with prebiotics

Before the main experiment, a pilot study was carried out to check optimal conditions for probiotics growth. Cultures were prepared with additional glucose (Alchem, Poland) 2% (w/v) a readily available carbon source for analyzed bacteria. The experiment was carried out in the 96-well cell culture plates. The number of replicants for each probiotic was six. Each well contained 5% bacterial inoculum (optical density, OD = 1). The bacterial suspensions of inoculum were prepared in 0.85% NaCl solution, from the colonies grown on the dedicated agar plates for 24 h (MRS: B1, B2, B3, B4, B6), (TSA: B5), (MRCB: B7). The optical density (600 nm) was measured every 2 h for 24 h with the use of a microtiter plate reader (SpectraMax® ID3 Multi-Mode Microplate Reader by Molecular Devices^®^) to check growth stimulation by a simple carbon source (Table 3).

**Table 3:** Identification of three bacterial strains described in this study.

Name	T bp	closest BLAST match in GenBank (NCBI) [Accession Number]	Similarity %	Classified as	Accession number in NCBI
CB1	1,427	*Carnobacterium divergens* [MN229536] *Carnobacterium divergens* [LC279606]	100% 99.93%	*Carnobacterium divergens*	OP804339
LB1	1,427	*Lactobacillus plantarum strain 3,356* [MT613640] *Lactobacillus plantarum strain 3,355* [MT613639]	99.93% 99.93%	*Lactobacillus plantarum*	OP804340
TH1	1,427	Acidipropionibacterium thoenii* strain 866 [MF564049] *Propionibacterium thoenii* * strain JCM 6435 [AB.729071]	99.79% 99.79%	*Acidipropionibacterium thoenii*	OP804341

*Based on NCBI and Genbank Data Acidipropionibacterium thoenii and Propionibacterium thoenii are the same Bacteria.

### The growth kinetics of synbiotics

The growth rate of 7 probiotics in the presence of 12 prebiotics was measured by using a microtiter plate reader (SpectraMax^®^ ID3 Multi-Mode Microplate Reader by Molecular Devices^®^) at 600 nm wavelength, every 2 h, for a period of 24 h. Bacteria with prebiotics were cultivated in 96-well microtiter plates, with each well filled with 110 μL of medium, 80 μL of prebiotic solution and 10 μL bacterial inoculum (1 McFarland equal to 3.0 × 10^^8^ bacterial colonies). The number of replicants (wells) for each probiotic was six.

### Statistical analysis

The one-way ANOVA test with *post hoc* Tukey’s test were used for the calculation of significant differences between the growth kinetics of each combination of probiotics with prebiotics as compared to the control (*p* ≤ 0.05) (Statistica 10.0; StatSoft).

## Results

### Optimization of growth conditions for probiotics

**Figure 1:**
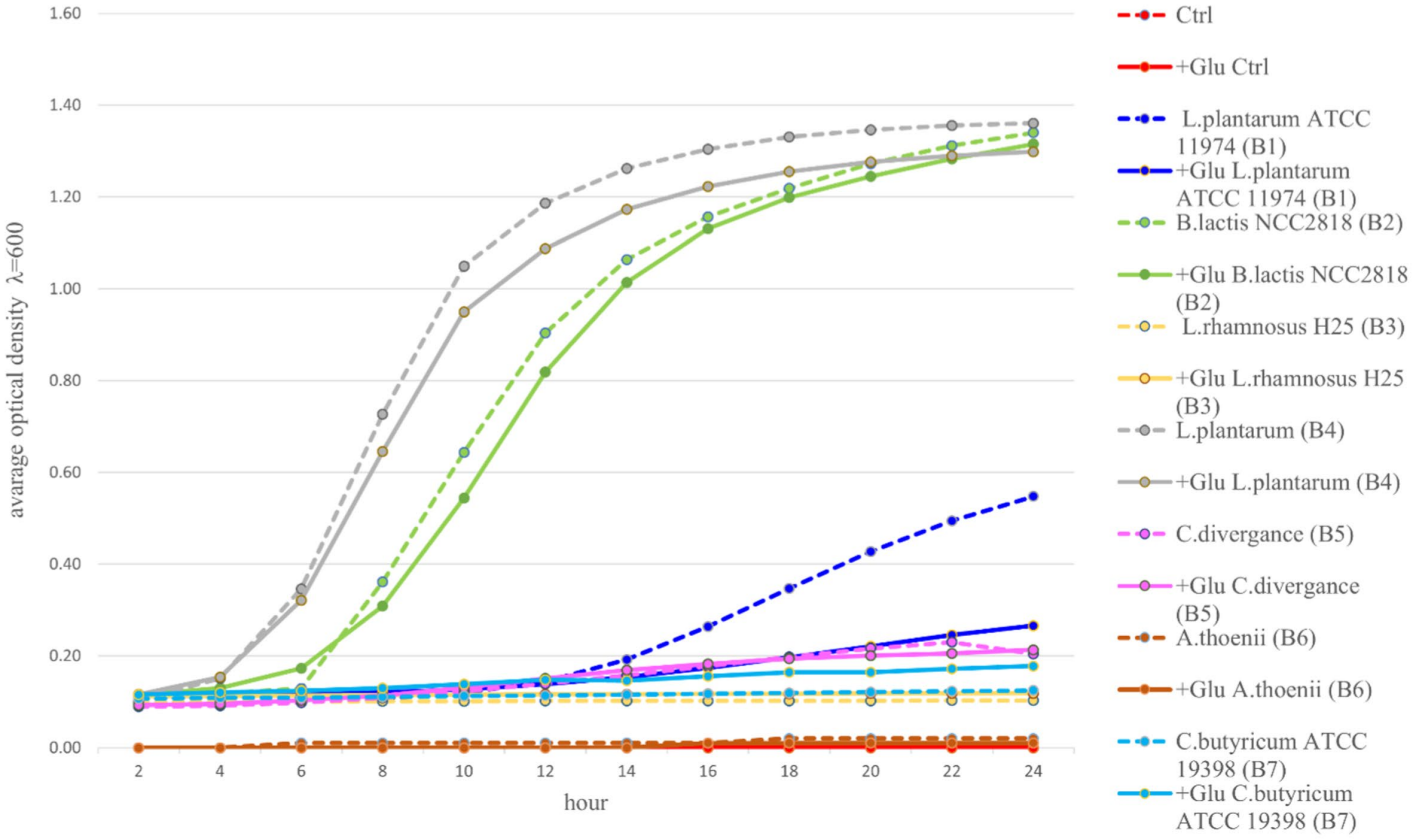
Growth kinetics of seven probiotic bacteria (B1-B7) in an appropriate medium with or without glucose for 24 h. (Ctrl, control; Glu, glucose).

Among the seven tested probiotic bacteria, the best growth in MRS medium and MRS medium enriched with glucose were observed for two tested strains: B2 (*B. lactis*) and B4 (*L. plantarum*). These bacteria entered the exponential growth phase (intensive growth) already after 2 h of incubation and reached the equilibrium phase in 14-16 h of incubation, with OD values greater than 1.0. In the case of three other strains: B3 (*L. rhamnosus* H25), B5 (*C. divergens*), and B7 (*C. butyricum* ATCC 19398), significantly lower growth was observed. These strains entered the phase of logarithmic growth as late as 12 h of incubation and reached the maximum growth on an average level between 0.1–0.3 OD. Bacterial strain B6 (*A. thoenii*) revealed only a minimal growth, lower than 0.1 OD. In this experiment, we used glucose as a control, as it is considered the preferred carbon source for most probiotic bacteria. Our results showed that the probiotics B1 (*L. plantarum* ATCC 11974), B2 (*B. lactis*) and B4 (*L. plantarum*) had higher growth rates without addition of glucose. In the case of probiotics: B3 (*L. rhamnosus* H25), B5 (*C. divergence*) and B6 (*P. thoenii*), there was no significant difference between the two microbial substrates. Only for probiotic B7 (*C. butyricum*), the growth of bacteria in the presence of glucose was higher than the rest of the prebiotics (Figure 1).

### Growth stimulation of probiotics by prebiotics

**Figure 2:**
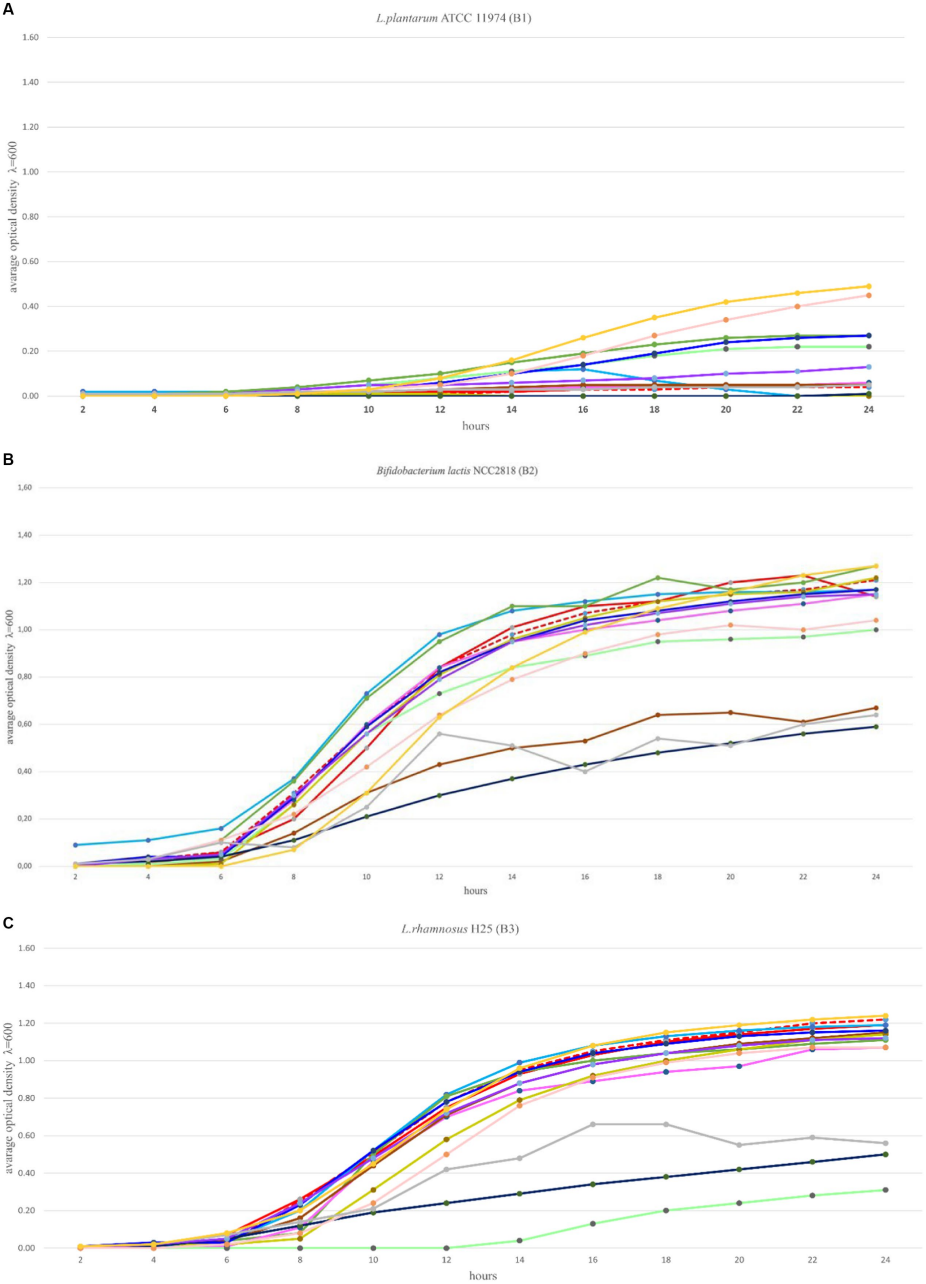

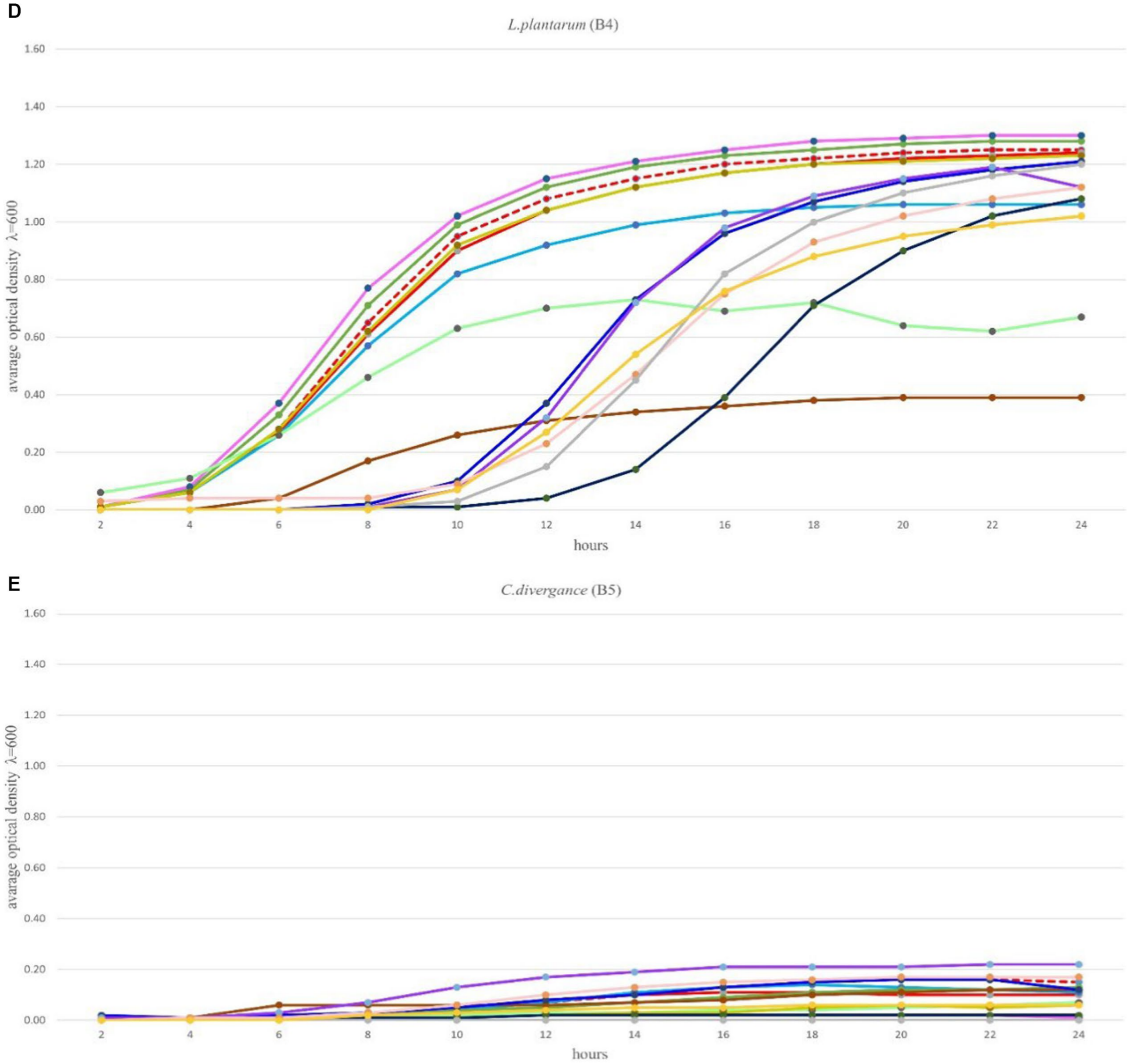

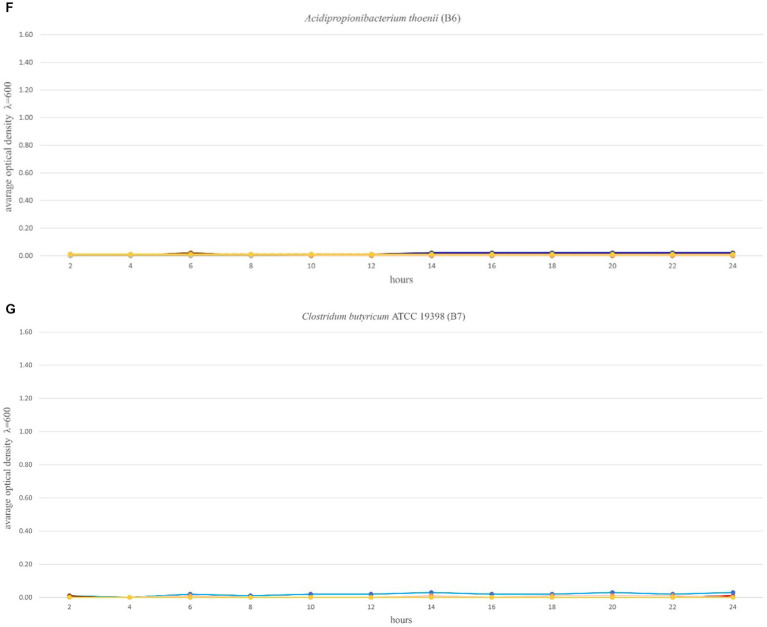
**(A–G)** Growth performance of seven probiotic bacteria (a–g, B1-B7) in the presence of 12 prebiotic compounds (P1-P12) for 24 h. Control (Ctrl) is marked in red color. Other colors represent a different prebiotic according to information presented in Table 1.

The optimal growth of the seven tested probiotics (B1-B7) in the presence of 12 prebiotic compounds (P1-P12) was tested every 2 h for a period of 24 h using a microplate reader (SpectraMax^®^ ID3). In accordance with Polak-Berecka et al. (2013) the probiotics were cultivated in microplates in media with prebiotics added, for a minimum of 48 h until the stationary phase was reached. In this study, the optimal growth of the probiotics was assessed during a 24 h time period. Growth ranges of the tested probiotics depended on the strain and the prebiotic present in the medium and ranged from 0–1.30 (Figures 2A–G). The highest growth values in the control variants (microbiological medium MRS and MRS enriched with glucose) and in the presence of the tested prebiotics (P1-P12) were observed for three strains: B2 (*B. lactis*), B3 (*L. rhamnosus*) and B4 (*L. plantarum*), for which the OD values ranged from 0 to 1.35 (Figures 2B,D). Strain B1 (*L. plantarum*) showed moderate growth both in the control medium (with glucose) and with prebiotics, ranging from 0 to 0.5. In the case of strains: B5 (*C. divergens*), B6 (*A. thoenii*) and B7 (*C. butyricum*), a much weaker growth was observed, both in the control and in the presence of all the 12 tested prebiotics, which ranged from 0 to 0.2 (Figures 2E–G). This was probably due to a generally poor growth rate of these strains in our study and/or the preference of CO_2_ conditions.

Several prebiotics specifically stimulated the growth of bacteria. The growth of probiotic strains: B1 (*L. plantarum* ATCC 11974) and B3 (*L. rhamnosus*) was stimulated by the presence of prebiotic P12 (astragalus polysaccharides) in the medium, after 8 h of incubation. The growth of strain B1 (*L. plantarum* ATCC 11974) was specifically stimulated by the prebiotic P10 (lentinus). The strains: B2 (*B. lactis*) and B3 (*L. rhamnosus*) were stimulated after 4 and 8 h of incubation by the presence of prebiotics P1 (Beta-glucan) and P12 (astragalus polysaccharides) in the media, respectively. The growth of strain B4 (*L. plantarum*) was stimulated by the prebiotics P2 (vegetable protein hydrolysate) and P3 (seaweed liquid extract) after 4 h of incubation, and the result was higher compared to the glucose-enriched control medium. In the case of strain B4 (*L. plantarum*), comparable growth was observed in the presence of the prebiotic P6 (raffinose). It’s significant to note that some of the studied prebiotics, including P5 (long chain inulin) and P8 (chitooligosaccharides obtained from snow crabs), inhibited the growth of the tested probiotics.

### Optimization of synbiotic formulations based on growth kinetics

**Figure 3:**
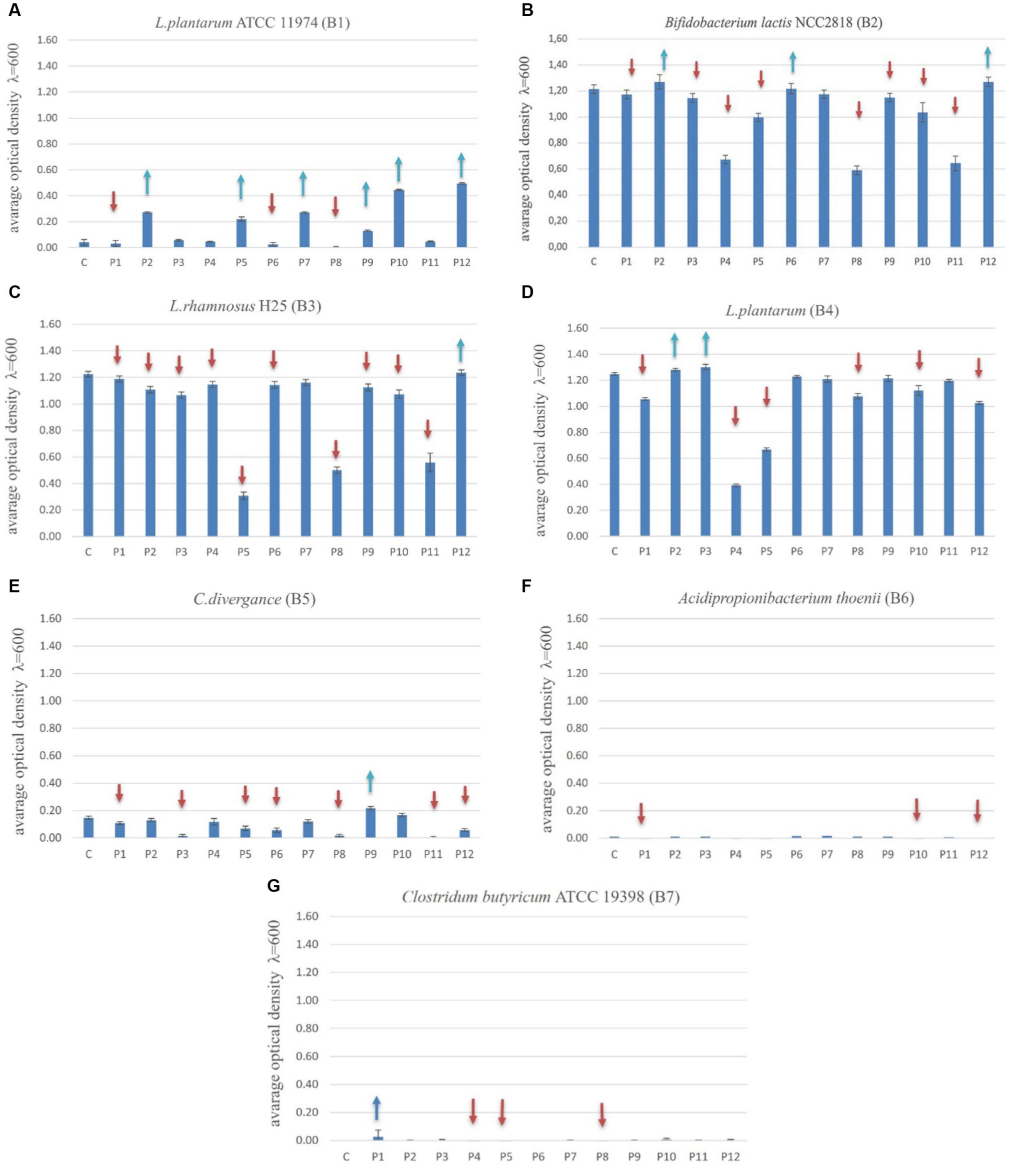
**(A–G)** the growth of probiotics (B1-B7) with prebiotics (P1-P12) after 24 h of their incubation. The one-way ANOVA with Tukey’s test as *post hoc* (Newman–Keuls) comparisons (*p* ≤ 0.05) (Statistica 10.0; StatSoft). Blue arrows show stimulation of the probiotic growth and red arrows show the inhibition of the probiotic growth compared to the control variant (C, Control with glucose).

The optimal overall growth of bacteria after 24 h incubation in the presence of prebiotics was observed for strains B2 (*B. lactis*), B3 (*L. rhamnosus*) and B4 (*L. plantarum*), ranging from 0 to 1.35. The strains B1 (*L. plantarum*) and B5 (*C. divergens*) had OD values ranging from 0 to 0.50 and 0 to 0.20 respectively, while the strains B6 (*A. thoenii*) and B7 (*C. butyricum*) had lower OD values in the range of 0 to 0.05. The “universal” strains prone to stimulation by the largest number of prebiotics were: B1 (*L. plantarum*) (stimulated by 6 prebiotics), B2 (*B. lactis*) (by 3 prebiotics) and B4 (*L. plantarum*) (stimulated by 2 prebiotics). The growth of strains B3 (*L. rhamnosus*) and B5 (*C. divergence*) was stimulated by only a single prebiotic. The “universal” prebiotics that significantly stimulated the growth of most of the bacterial strains were: P2 (vegetable protein hydrolysate) and P12 (astragalus polysaccharides) (Figures 3A–G).

Statistical analysis revealed that over 24 h, the growth of B1 strain (*L. plantarum*) was increased by 14.3% with prebiotic P2 (vegetable protein hydrolysate) compared to glucose (carbon basic source), 14% with P5 (long chain inulin) compared to glucose, 14% more with P7 (galactooligosaccharides, GOS) compared to glucose, and 5.6% more with P9 (saccharicter-penin), P10 (lentinus) and P12 (astragalus) compared to glucose (Figures 3A–G). After 24 h of incubation, the growth rate of strain B2 (*B. lactis*) was stimulated 2.5% more by three prebiotics: P2 (vegetable protein hydrolysate), P6 (raffinose) and P12 (astragalus) compared to the control glucose. The growth of B3 (*L. rhamnosus*) was stimulated only by P12 (astragalus) and was equal to the growth in the presence of a control glucose. Strain B4 (*L. plantarum*) was stimulated by two prebiotics: P2 (vegetable protein hydrolysate) and P3 (liquid seaweed extract). The highest effect of growth stimulation by prebiotics in this experiment was observed in the case of the B1 strain (*L. plantarum*). However, the growth of this bacteria was low in general. For B1, the statistical analysis revealed significant growth stimulation by as many as four prebiotics: P2 (vegetable protein hydrolysate), P5 (long chain inulin), P7 (GOS) and P9 (saccharicter-penin). The strain B2 (*B. lactis*) showed a significantly higher growth with 2 prebiotics: P2 (vegetable protein hydrolysate) and P12 (astragalus). It was a 2.5% higher proliferation than in the presence of glucose as the reference carbon source (control).

## Discussion

*In vitro* tests allow to examine the potential interactions between the two components. The major purpose is to supply safe and beneficial bacteria acting as probiotics, and/or to add prebiotics that would support establishing the right habitat for microorganisms in the animal gut. In this study, we selected 2 probiotics and 2 compatible prebiotics considered optimal to formulate the synbiotics for *in ovo* injections in the chicken trials.

Most of the Lactobacilli strains are commercially available and show a significant effect on poultry (Bogucka et al., 2019). *Lactobacillus animalis*, *L. rhamnosus* and *L. reuteri* were shown to increase chicken feed conversion ratio (FCR) and lower the number of pipped eggs when they were injected *in ovo* into chickens (Li et al., 2021). In another study, this group of probiotics was shown to decrease the level of *Campylobacter* spp. in the treated chickens (Smialek et al., 2018). According to (Succi et al., 2017) the resistance and survivability of *L. rhamnosus* under simulated GI conditions are increased by pre-cultivating the probiotic using specific sources of inulin, lactulose, mannitol, or sorbitol. Only *L. rhamnosus* H25 demonstrated greater compatibility with the prebiotics examined here, making it a recommendation for synbiotic studies between all strains which belong to the Lactobacillus family in our work (*L. plantarum* ATCC 11974 – B1, *L. plantarum* – B4, *L. rhamnosus* H25 – B3). This conclusion was drawn from the results of the bacterial growth analysis (based on absorbance), which is an extremely important element in determining the preparation of a synbiotic and scaling up production.

A combination of *Lactobacillus* and *Bifidobacterium* probiotics could inhibit *S. typhimurium* intestinal colonization in infected chickens by competitive exclusion or cytokine production mechanisms (El-Sharkawy et al., 2020). Also, according to (Hajati and Rezaei, 2010), *Bifidobacterium* has an antibacterial impact because it may prevent possible intestinal infections, *Bifidobacterium bifidum*, *B. animalis*, *B. longum* and *B. infantis* were associated with an increased (*p* < 0.01) in body weight and weight gain in all treated poultry groups (increases of at least 5.38 and 8.27%). *In ovo* injection results also showed that total fecal coliform counts were numerically reduced in the *in ovo*-inoculated groups (Abdel-Moneim et al., 2020). *Bifidobacterium lactis* (B2) in our study was shown to be a strong candidate for future trials on potential synbiotics. This was determined after analyzing bacterial growth (measured using absorbance), which is a crucial step in assessing whether the chosen bacterial strain will proliferate quickly during the development of a synbiotic on a large scale during production.

According to (Smialek et al., 2018) the presence of *C. divergens* in the gastrointestinal tract (GIT) of chickens resulted in a lower contamination level in the avian environment and better sanitary characteristics of the tested flock. In our study, *C. divergens* (B5) started to proliferate late, after at least 14 h of culture. It can be considered a slow-growing probiotic and may be further explored as a potential candidate in synbiotic formulations for the treatment of undeveloped GIT (early life), where the fast over-proliferation of a single strain could lead to the harmful, dysbiosis effect.

The mechanism by which *C. butyricum* promotes growth performance in chickens as a probiotic is considered highly complex. According to Yang et al. (2012), the higher serum concentrations of IgA and IgM found in chickens fed with *C. butyricum* are linked with improved immunological function. Moreover, *C. butyricum* decreased (*p* < 0.05) the level of *Escherichia coli* in cecal contents (Yang et al., 2012). Probiotics may induce non-specific immunomodulatory effects that are thought to be advantageous in situations needing activation of defensive mechanisms (e.g. by stimulating T-cells). There are few studies examining the potential of *Propionibacteria* as probiotics. Based on the earlier mammalian studies, this probiotic may attach to epithelial cells, and live in acidic conditions and the presence of bile salts, with a modest antibacterial activity against *E. coli* (Campaniello et al., 2015). In our study, *A. thoenii* (B6) and *C. butyricum* (B7) were characterized by slow growth rates and a late entry to the proliferation phase, after 14 h of incubation. In this study, the optimal growth rate of *C. butyricum* (B7) with different prebiotics was tested for the first time. We found that beta-glucan (P1) may be a good prebiotic candidate to study the synergistic effect with this bacteria.

Prebiotics are defined as fermentable oligosaccharides that specifically increase the development of beneficial bacteria in the intestine and help the establishment of a healthy microbiota. In this study, Beta-glucan (P1) stimulated the growth rate of fast and moderate-growing probiotics such as *L. plantarum ATCC 11974* (B1), *B. lactis* (B2), and *L. rhamnosus H25* (B3). Beta-glucan extracted from the *Laminaria* spp. was shown to increase body weight and was associated with an increase in the number of lactobacilli and bifidobacteria in chicken faeces (Bednarczyk et al., 2016). Inulin was also assessed as a prebiotic in this study, both long-chain and standard. Only long-chain inulin stimulated the growth of *L. plantarum* ATCC while the two types of inulin mostly inhibited the growth rate of the remaining probiotics. In other studies, researchers mainly found that inulin was effective in enhancing and stimulating the growth of probiotics (Bucław, 2016; Kozłowska et al., 2016). Raffinose in our study stimulated the growth of *B. lactis* (B2). Raffinose was tested earlier as a prebiotic and synbiotic component for poultry, including an *in ovo* study (Stadnicka et al., 2020). It was shown to increase villus surface area, improve gut health by promoting the survival of probiotics and limiting the presence of potentially pathogenic bacterial populations such as *E. coli*, or led to improved micro vascularization and muscle development *in ovo* (Bogucka et al., 2022). Raffinose was shown to decrease the level of Clostridium’s relative abundance (Pacifici et al., 2017). In this study, GOS stimulated the growth rate of *L. plantarum* (B1) higher than the control but for the rest of the probiotics, it was like the control. GOS is described in several different studies on poultry prebiotics and is found to increase feed intake. *In ovo* delivery of GOS may cause natural Eimeria infection resulting in less severe intestinal lesions and oocyst excretion, and this had a good impact on the productive features of chickens (Angwech et al., 2019).

According to (Trojanová et al., 2009) glucose and raffinose as prebiotics utilized in probiotic culture affected the cell morphology of probiotic strains of *Bifidobacterium*. The description of *Bifidobacterium*’s preferential consumption of diverse saccharides may aid in the creation of new synbiotic preparations and growth media for *bifidobacteria*. In another study (Bevilacqua et al., 2016), lactulose prebiotic was successfully utilized to promote *bifidobacterium*, leading to a better growth index for this probiotic compared to glucose as a carbon source. Therefore, lactulose could be a good ingredient for a synbiotic combination. The use of prebiotics such as GOS and lactulose as an alternative to monosaccharides, may stimulate the growth of probiotic *Lactobacillus* strains and increase their survival through the gastrointestinal system (Hernandez-Hernandez et al., 2012). This study suggests that the most suitable synbiotic combinations are B1 (*Lactoplanus plantarum* ATCC 11974) and B2 (*Bifidobacterium lactis* NCC2818) in combination with the prebiotics astragalus (P12) and vegetable protein hydrolysate (P2).

## Conclusion

This study outlines a useful methodology for the selection of suitable probiotic and prebiotic combinations to be further tested as candidate synbiotics. The results show that the newly characterized probiotics and natural prebiotic ingredients isolated from natural sources, can considerably enhance the development of probiotics, prebiotics and synbiotic formulations for “tailored” applications. In particular, high growth stimulation was observed for several candidate synbiotic combinations, namely the three tested probiotics (*B. lactis* (B2), *L. rhamnosus* (B3) and *L. plantarum* (B4)) in the presence of specific prebiotics (terrestrial plant and marine algae derived extracts, and astragalus polysaccharides). Such combinations are recommended for the further investigation in poultry trials. Importantly, these probiotics and prebiotics can be considered as good candidates for *in ovo* dosage testing trials followed by the post-hatch rearing. The safety of the selected probiotics is widely acknowledged and they function as registered and approved probiotics in the EU. The selected prebiotics are fully soluble in the injection solution, which is a prerequisite for *in ovo* applications. From an economical point of view, further investing in the optimization of new formulations for *in ovo* applications is justified. It potentially allows for the delivery of immune modulators and natural compounds that influence gut development at minimal doses at the earliest possible embryonic stages, thus reducing the cost of further treatments (milligrams of inoculates vs. kg of feed additives). The *in vitro* stage is suggested as a requirement prior to *in vivo* trials. It has the potential to be widely utilized by producers of natural compounds to meet the needs of the *in ovo* applications market.

## Data availability statement

The datasets presented in this study can be found in online repositories. The names of the repository/repositories and accession number(s) can be found at: https://www.ncbi.nlm.nih.gov/, HQ259724.1; https://www.ncbi.nlm.nih.gov/, MT613640.1; https://www.ncbi.nlm.nih.gov/, AB729071.1.

## Author contributions

KS and KH conceived and conceptualized the work and supervised the project. NA drafted the article. NA, DT, and KH carried out the experiments and analyzed data. JO’S, AW, KG, and CR optimized and provided novel (non-commercial) bioactive formulations for the trials. KS, KH, DT, JO’S, AW, KG, and CR critically revised the article. All authors listed have made substantial, direct, and intellectual contributions to the work and approved it for publication.
