# Supplementary material for: Evaluation of probiotic growth stimulation using prebiotic ingredients to optimize compounds for in ovo delivery
Source: Front Microbiol. 2023 Sep 22;14:1242027. doi: 10.3389/fmicb.2023.1242027 (PMC10556452; doi:10.3389/fmicb.2023.1242027)
Supplement: Supplementary file 1 [file Data_Sheet_1.pdf]

Supplementary Material

Supplementary Figure A. Results showing optimal growth of probiotics with prebiotics: 0-4 h (fast growth), 4-12 h (moderate growth) and 12-24 h (slow growth).

**Probiotics**	**Prebiotics**																																						
	Beta Glucan			Vegetable Protein hydrolysate			Liquid seaweed extract			Standard inulin			Long chain inulin			Raffinose			GOS			Snow crab-derived chitooligosaccharides			Saccharicter-penin			Lentinus			Mannan oligosaccharides			Astragalus Polysaccharide		
	>4h	>12	>24	>4h	>12	>24	>4h	>12	>24	>4h	>12	>24	>4h	>12	>24	>4h	>12	>24	>4h	>12	>24	>4h	>12	>24	>4h	>12	>24	>4h	>12	>24	>4h	>12	>24	>4h	>12	>24
***Lactobacillus plantarum* ATCC 11974**						×									×						×						×			×						×
***Bifidobacterium lactis* NCC2818**	×	×	×	×		×		×																							×					×
***Lactobacillus rhamnosus* H25**		×	×	×	×	×		×																										×		×
Lactobacillus plantarum				×	×	×	×	×	×							×																				
Carnobacterium divergens																											×			×						

Acidipropionibacterium thoenii																																				
***Clostridium butyricum* ATCC 19398**			×																																	×
